# Supplementary material for: Multi‐dataset identification of innovative feature genes and molecular mechanisms in keratoconus
Source: J Cell Mol Med. 2024 Sep 19;28(18):e70079. doi: 10.1111/jcmm.70079 (PMC11412914; doi:10.1111/jcmm.70079)
Supplement: Supplementary file 2 — Material S2. [file JCMM-28-e70079-s002.zip › 7.GSEA analysis/2.ATOH7/KEGG_ETHER_LIPID_METABOLISM.html]

Details for gene set KEGG\_ETHER\_LIPID\_METABOLISM[GSEA]

|  || Dataset | ATOH7.group.cls#h\_versus\_l |
| Phenotype | group.cls#h\_versus\_l |
| Upregulated in class | h |
| GeneSet | KEGG\_ETHER\_LIPID\_METABOLISM |
| Enrichment Score (ES) | 0.55153733 |
| Normalized Enrichment Score (NES) | 1.4147736 |
| Nominal p-value | 0.087128714 |
| FDR q-value | 0.29341868 |
| FWER p-Value | 0.892 |
Table: GSEA Results Summary

  

Fig 1: Enrichment plot: KEGG\_ETHER\_LIPID\_METABOLISM      
 Profile of the Running ES Score & Positions of GeneSet Members on the Rank Ordered List

  

| SYMBOL | TITLE | RANK IN GENE LIST | RANK METRIC SCORE | RUNNING ES | CORE ENRICHMENT || 1 | LPCAT2 | na | 270 | 0.638 | 0.1294 | Yes |
| 2 | LPCAT1 | na | 627 | 0.516 | 0.2301 | Yes |
| 3 | CHPT1 | na | 1386 | 0.399 | 0.2940 | Yes |
| 4 | PLA2G7 | na | 1426 | 0.394 | 0.3776 | Yes |
| 5 | PLA2G1B | na | 1671 | 0.371 | 0.4504 | Yes |
| 6 | PLA2G2C | na | 2003 | 0.344 | 0.5149 | Yes |
| 7 | PLA2G2A | na | 2876 | 0.287 | 0.5515 | Yes |
| 8 | ENPP2 | na | 7554 | 0.129 | 0.4446 | No |
| 9 | PAFAH1B2 | na | 8114 | 0.116 | 0.4536 | No |
| 10 | ENPP6 | na | 11529 | 0.059 | 0.3678 | No |
| 11 | PAFAH1B3 | na | 12669 | 0.044 | 0.3444 | No |
| 12 | PLD1 | na | 14651 | 0.024 | 0.2925 | No |
| 13 | PLA2G4A | na | 15227 | 0.019 | 0.2801 | No |
| 14 | PLA2G6 | na | 15813 | 0.014 | 0.2663 | No |
| 15 | PLD2 | na | 17242 | 0.002 | 0.2256 | No |
| 16 | PAFAH2 | na | 17926 | -0.003 | 0.2066 | No |
| 17 | PLA2G12A | na | 18221 | -0.006 | 0.1994 | No |
| 18 | PAFAH1B1 | na | 18249 | -0.006 | 0.2000 | No |
| 19 | PLA2G2E | na | 18403 | -0.008 | 0.1972 | No |
| 20 | LPCAT4 | na | 20066 | -0.022 | 0.1541 | No |
| 21 | AGPS | na | 20334 | -0.025 | 0.1518 | No |
| 22 | JMJD7-PLA2G4B | na | 23573 | -0.063 | 0.0722 | No |
| 23 | PLA2G3 | na | 24134 | -0.071 | 0.0714 | No |
| 24 | PLA2G5 | na | 25954 | -0.103 | 0.0411 | No |
| 25 | PLA2G10 | na | 27060 | -0.127 | 0.0366 | No |
| 26 | PLA2G4E | na | 27235 | -0.132 | 0.0599 | No |
| 27 | PLA2G2F | na | 27530 | -0.139 | 0.0813 | No |
| 28 | PLA2G2D | na | 28487 | -0.164 | 0.0891 | No |
| 29 | PLA2G12B | na | 33562 | -0.422 | 0.0336 | No |
Table: GSEA details [plain text format]

  

Fig 2: KEGG\_ETHER\_LIPID\_METABOLISM      
 Blue-Pink O' Gram in the Space of the Analyzed GeneSet

  

Fig 3: KEGG\_ETHER\_LIPID\_METABOLISM: Random ES distribution      
 Gene set null distribution of ES for **KEGG\_ETHER\_LIPID\_METABOLISM**
